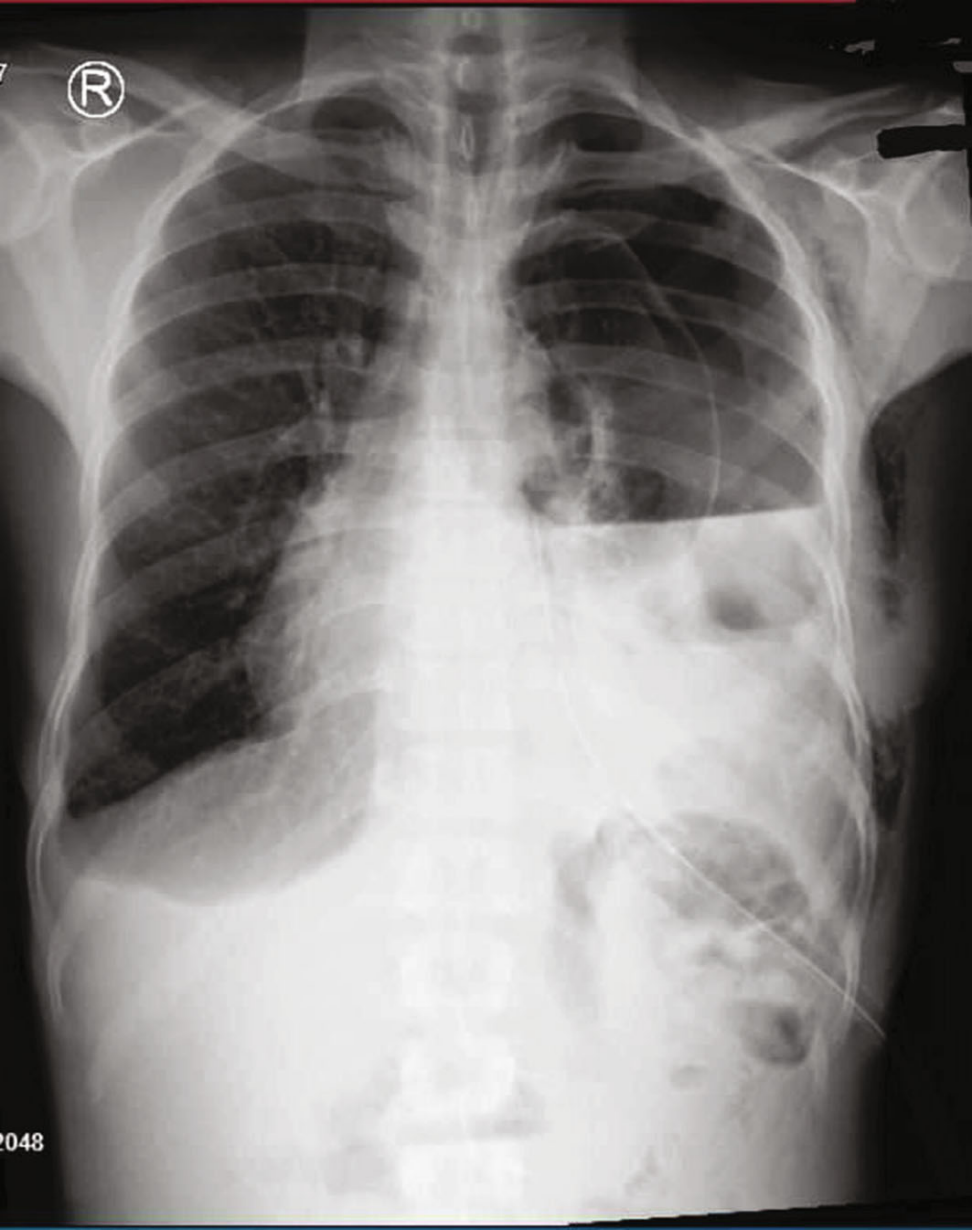# Surgical Approach in Management of Posttraumatic Diaphragmatic Hernia: Thoracotomy versus Laparotomy

**DOI:** 10.1155/2020/6694990

**Published:** 2020-12-05

**Authors:** Ahmed Shabhay, Pius Horumpende, Zarina Shabhay, Sjef G. Van Baal, Ester Lazaro, Kondo Chilonga

**Affiliations:** ^1^Department of General Surgery, Kilimanjaro Christian Medical University College (KCMUCo), P.O. Box 2240 Moshi, Tanzania; ^2^Department of General Surgery, Kilimanjaro Christian Medical Centre (KCMC), P.O. Box 3010 Moshi, Tanzania; ^3^Institute of Infectious Diseases and Research, Lugalo Military College of Medical Sciences (MCMS) and General Military Hospital (GMH), P.O. Box 60000 Dar es Salaam, Tanzania; ^4^Kilimanjaro Clinical Research Institute (KCRI), P.O. Box 2236 Moshi, Tanzania; ^5^Muhimbili Orthopedic Institute (MOI), P.O. Box 65474 Dar es Salaam, Tanzania; ^6^ZGT Academy, Hospital Group Twente, Almelo/Hengelo, Netherlands; ^7^Cardiff University, Cardiff, UK; ^8^Department of Radiology, Kilimanjaro Christian Medical Centre (KCMC), P.O. Box 3010 Moshi, Tanzania

## Abstract

Breach in diaphragmatic musculature permits abdominal viscera to herniate into the thoracic cavity. Time of presentation and associated injuries determines the surgical approach in management. This case report sets to highlight the challenges in clinical diagnosis, radiological interpretation, and surgical management approaches of posttraumatic diaphragmatic hernia. We report a case of a 43 years old male who was diagnosed with traumatic diaphragmatic hernia 6 months post blunt thoracoabdominal trauma due to motor traffic accident. He was initially diagnosed with haemothorax, drained with an underwater thoracostomy tube, and discharged. He continued to experience on and off chest pain worsening postfeeding, difficulty in breathing and abdominal pain for the next six months until his eventual diaphragmatic hernia diagnosis. He was scheduled for an elective thoracotomy. A left posterolateral thoracic over the 7^th^ intercostal space incision was used. Intraoperatively, the stomach, left lobe of liver, part of transverse colon, small bowel, and omentum had herniated into the thoracic cavity adhering into thoracic viscera and wall. Adhesiolysis was done, and abdominal organs reduced into abdominal cavity. Rent was closed by interrupted Prolene sutures reinforced with a mesh. In patients with delayed presentation of diaphragmatic hernia post blunt thoracoabdominal injury without associated intra-abdominal visceral injury, we recommend the thoracic diaphragmatic repair approach as long-standing herniated bowels might adhere with thoracic cavity walls or viscera. In such cases, adhesiolysis and rent repair is easier through thoracotomy.

## 1. Introduction

Diaphragmatic hernia post blunt thoracoabdominal trauma was described by Sennertus as early as 1541 [1, 2]. Three decades later Pare demonstrated diaphragmatic hernias in postmortem autopsies with a history of blunt trauma [1]. Traumatic diaphragmatic rupture is a rare, potentially life-threatening condition due to blunt or penetrating thoracoabdominal trauma often misdiagnosed at emergency care settings [3, 4]. In acute emergency setup, patients with diaphragmatic rupture, laparotomy is the recommended approach as inspection of associated intra-abdominal viscera injury is done [5–8]. In cases with delayed presentation, thoracotomy is the best approach as it is easier to reduce abdominal contents, release of any bowels adhesions, and repair the rent [2, 6–8]. In this case report, we set out to show the diagnostic challenges and surgical approaches in management of diaphragmatic hernias.

## 2. Case Presentation

A 43-year-old man was involved in a high energy motor traffic accident and sustained blunt thoracoabdominal injury. At the emergency department, his vitals were stable with oxygen saturation of 96% with room air. He had reduced air entry on the left side of the chest. His chest X-ray showed blunted costophrenic and cardio phrenic angle. He was diagnosed with haemothorax, admitted at General Surgical ward, and a thoracostomy under water tube inserted. Approximately 2000 ml of haemolysed blood was drained over 7 days. Apart from minor bruises, he had no other diagnosed accompanied injuries. He was discharged uneventfully. Two weeks later, he presented at the Emergency Department with a history of difficulty in breathing, left-sided chest pain, and left upper quadrant abdominal pain. A chest X-ray was done and revealed a blunted coastal phrenic angle. A recollected haemothorax was reached as a diagnosis. An underwater seal thoracostomy tube was reinserted, and a total of approximately 1000 ml of haemolysed blood was drained over 7 days, and he was discharged.

For the next six months postdischarge, during subsequent visits at the General Surgical outpatient clinic, the patient complained of on and off episodes of left-sided chest pain worse after eating, episodes of difficulty in breathing, and abdominal pain. A control chest X-ray was then done and revealed a homogenous opacity, elevated hemidiaphragm, an irregular diaphragmatic outline, and atelectasis of the left lung, all suggestive of diaphragmatic rupture (Figure 1). CT scan of chest revealed elevation of the left diaphragmatic copula reaching DV6/7 level associated with relaxation atelectasis of the left lower lobe with preserved vascular and bronchiolar tree distribution.

The patient was scheduled for an elective thoracotomy. A left posterolateral thoracotomy incision above the 7^th^ intercostal space was used. Intraoperatively, we found an 8 by 8 cm rent in the posterolateral part of the left dome with herniation of part of transverse colon, left lobe of liver, stomach, part of omentum, and small bowel with pericardial tear 4 by 5 cm exposing coronary vessels. There were multiple adhesions of the abdominal visceral organs on the walls of thoracic cavity. The left lung was adherent to the anterior thoracic wall. Adhesiolysis done and intestinal contents were mobilized back into the abdominal cavity. The rent was repaired with interrupted Prolene sutures and mesh laid over repaired defect. He was discharged two weeks later, and his follow-up was uneventful.

## 3. Discussion

Diaphragmatic rupture posttrauma was described as early as 1541 by Sennertus and Pare in 1579 in postmortem autopsies [9, 10]. First diaphragmatic hernia repair was reported by Riolfi in 1886 and Naumman in 1888 [1]. It occurs up to 5% of admitted trauma patients in Hospitals [4]. Rise in motor traffic accidents has led to its increased incidence [1, 5]. There are no reliable data on the incidence of diaphragmatic rupture [1]; however, its incidence ranges from 0.8% to 5% with less than 2.7% of cases diagnosed within four months postinjury [5]. The commonest etiology is blunt trauma [9]. It still remains a diagnostic challenge requiring a high index of suspicion [1, 9]. Diagnosis is delayed and will often be diagnosed once emergent conditions ensue or as an incidental finding during laparotomy [5, 11]. It is now being diagnosed more early due to the advancement of imaging modalities and awareness of medical stuff [11]. Spontaneous healing of diaphragmatic rupture has not been reported [1]. Once the diagnosis has been reached, surgery is way forward.

In this case, the patient's initial chest X-rays, which features suggestive of diaphragmatic rupture, i.e., elevation of left hemidiaphragm, an irregular diaphragmatic outline, and left lung atelectasis, were masked by homogenous opacification due to haemothorax. CT scan examinations were not routine investigations at our and relatively expensive. Patient was clinically stable with no clinical indication of advanced radiological investigation. Diaphragmatic rupture was not suspected in the acute setting as associated injuries masked his presentation. These factors together with a low clinical index of suspicion lead to the delay in diagnosis.

Surgical approach in management depends on the time of presentation, location of associated lesions, and surgeons experience [6]. Both thoracotomy and laparotomy approaches can be used in the repair of diaphragmatic hernias [6]. In acute settings, in patients with combined thoracic and abdominal viscera injury, a combined thoracic and abdominal cavity exploration is done [12]. In diaphragmatic hernia repair, with accompanied abdominal cavity, viscera injury laparotomy is the gold standard approach [2, 4, 10, 11]. However, in delayed presentation cases, long-standing herniated bowels tend to form adhesions with intrathoracic viscera [6]. In these cases, thoracotomy is the best approach [1, 2, 9, 11], as release of adhesions of intra-abdominal viscera in the thoracic cavity is difficult in laparotomy. It is easier to reduce herniated contents and repair of diaphragmatic hernia through thoracotomy when there is no accompanied intra-abdominal injuries [5, 9, 11]. However, some surgeons still advocate for the abdominal approach in long standing cases with adhesions, and thoracic approach in acute cases as long as intra-abdominal viscera injury has been ruled out [6]. Some surgeons would prefer the abdominal approach in delayed presentation cases as resection, and primary anastomosis of gangrenous incarcerated bowels is easier from the abdominal cavity [12]. There is no consensus on agreed approach for both acute and long-standing diaphragmatic hernias [6]. The choice of approach differs from authors and might be influenced by surgeons' preferences or center the procedure is being performed [12]. Silva et al. observed laparotomy as the most common approach used in acute presentation cases and thoracotomy in delayed presentation cases [6]. The best approach is determined by presence of associated injuries, center procedure being performed, and surgeons' expertise [12]. In our center, delayed presentation of traumatic diaphragmatic hernias, thoracic approach is preferred as rent repair with bowels adhesiolysis is done under direct visualization. Our patient had intra-abdominal organs adhering to thoracic wall and viscera, with atelectatic left lung adhering to anterior thoracic wall. Adhesiolysis of affected viscera was performed with relative ease and safety through the thoracic approach.

## 4. Conclusion

A high index of suspicion is required in diagnosing diaphragmatic rupture post blunt thoracoabdominal trauma in acute settings. Accompanied pathologies, i.e., hemopneumothorax opacification, might mask diaphragmatic rupture chest X-ray features. Trauma CT scan chest is superior in diagnosis of diaphragmatic rupture versus conventional X-ray examinations. Choice of surgical approach in management of traumatic diaphragmatic hernia is determined by the presence of associated injuries, center procedure being performed, and surgeons' experience or preferences. In the absence of thoracic or abdominal viscera injuries, either thoracotomy or laparotomy can be employed depending on surgeon's preference or expertise. However, in our settings, we opine in delayed diaphragmatic hernias, thoracotomy is the preferred approach as adhering bowels adhesiolysis on thoracic viscera can safely be performed, and rent repair is easier.

## Figures and Tables

**Figure 1:**